# Optimization of an in vitro bilayer model for studying the functional interplay between human primary retinal pigment epithelial and choroidal endothelial cells isolated from donor eyes

**DOI:** 10.1186/s13104-019-4333-x

**Published:** 2019-05-30

**Authors:** Karthikka Palanisamy, Coral Karunakaran, Rajiv Raman, Subbulakshmi Chidambaram

**Affiliations:** 1 R.S. Mehta Jain Department of Biochemistry and Cell Biology, KBIRVO, Vision Research Foundation, Chennai, India; 2 School of Chemical and Biotechnology, SASTRA University, Thanjavur, India; 3 Department of Vitreo-Retinal Services, Medical Research Foundation, Chennai, India; 4 Department of Biochemistry and Molecular Biology, Pondicherry University, Kalapet, Puducherry India

**Keywords:** Bilayer model, FITC dextran permeability, hRPE, hCEC, VEGF

## Abstract

**Objective:**

The microenvironment of outer retina is largely regulated by retinal pigment epithelium (RPE) and choroid. Damage to either of these layers lead to the development of age related macular degeneration (AMD). A simplified cell culture model that mimics the RPE/Bruch’s membrane (BM) and choroidal layers of the eye is a prerequisite for elucidating the molecular mechanism of disease progression.

**Results:**

We have isolated primary retinal pigment epithelial cells (hRPE) and human primary choroidal endothelial cells (hCEC) from donor eyes to construct a bilayer of hCEC/hRPE on transwell inserts. Secretion of VEGF in the insert grown bilayer was significantly higher (22 pg/ml) than hCEC monolayer (3 pg/ml). To mimic the disease condition the model was treated with 100 ng/ml of VEGF, which increased the permeability of bilayer for 20 kDa FITC dextran while addition of bevacizumab, a humanized anti-VEGF drug, reversed the effect. To conclude the transwell insert based human primary hCEC/hRPE bilayer model would be an ideal system for studying the disease mechanisms and the crosstalk between RPE and choroid. This model will also be useful in screening small molecules and performing drug permeability kinetics.

## Introduction

**Fig. 1:**
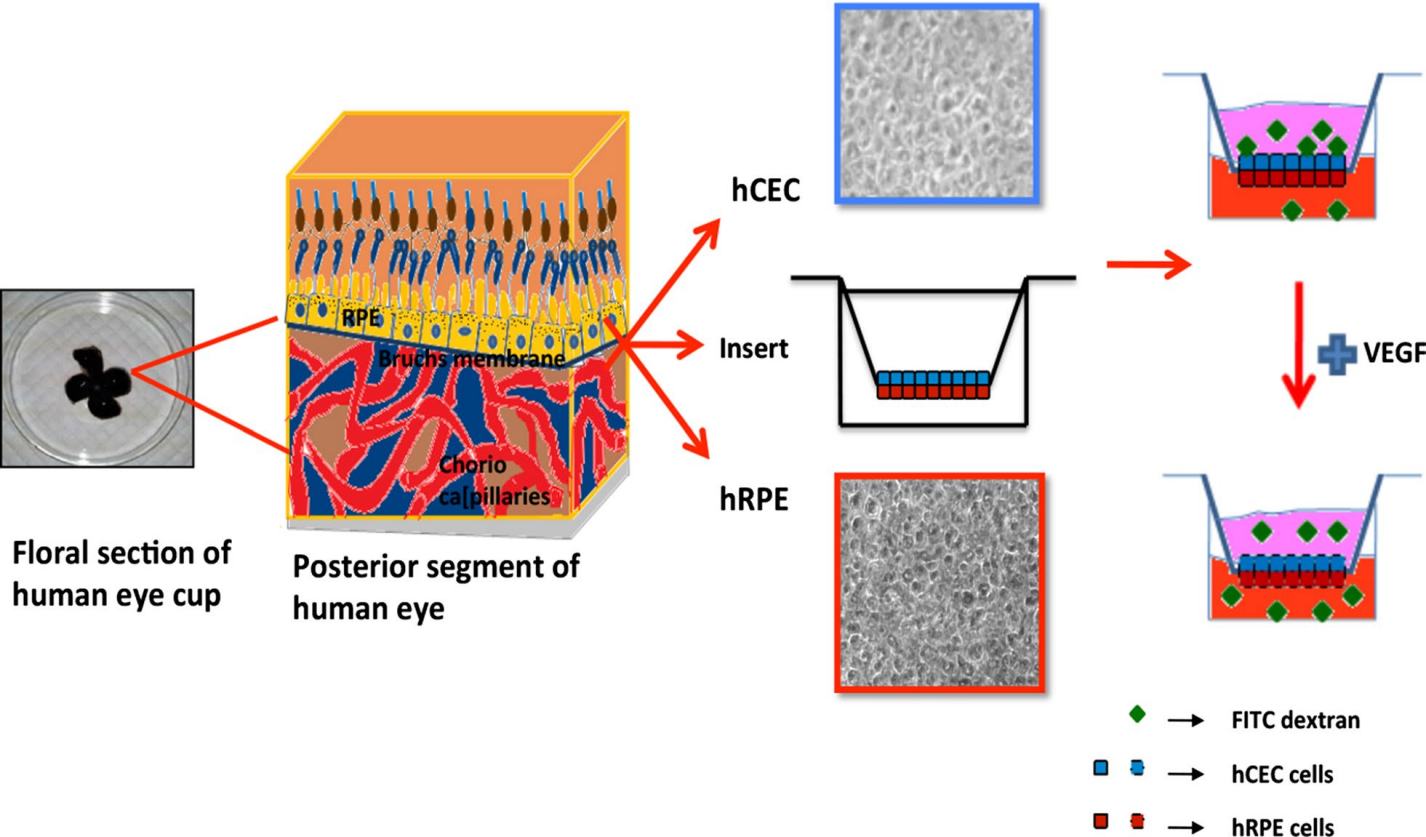
Schematic diagram of construction of bilayer model to mimic RPE/BM/choroid. The primary retinal pigment epithelial and choroidal endothelial cells were isolated from donor eyes. The primary cells were grown on either side of the transwell insert. To create the pathological milieu, these bilayer cells were treated with VEGF and their barrier permeability was checked using FITC-dextran conjugate

Retinal pigment epithelial (RPE) cells form the outer blood retinal barrier (oBRB) and control the transport of material from choroid. Posterior to RPE lies the Bruch’s membrane (BM) that forms the biochemical barrier controlling pathological events like choroidal neovascularization (CNV) [1]. RPE and choroid are mutualistic in function and any damage in one of the layers may result in the dysfunction of both [2]. The breakdown of RPE/BM/choroid leads to vascular leakage and accumulation of fluid in the extracellular space causing macular edema and eventually affects the visual acuity in age related macular degeneration (AMD). Lack of proper methodology to study RPE barrier in vivo is impeding the research on RPE/BM/choroid [3]. Besides, already existing in vitro models of RPE/BM/choroid using ARPE19 cell line and HUVEC [4–6] or using hRPE and primate choroid endothelial cell line (RF-6A) [7] might not entirely replicate the barrier function of in vivo retinal epithelial and choroid microvascular endothelial cells. Recently, Cao et al., was successful in culturing hRPE on human BM as ex vivo cultures for measuring changes in RPE atrophy [8]. Apart from in vitro and ex vivo cultures, few co-culture studies investigating synergistic role of retinal epithelial and endothelial cells have also been reported [9–12]. However, all co-cultures established so far have utilized immortalized ARPE19 cells and RF-6A or dermal microvascular endothelial cells. Thus, in this study a novel bilayer model using hCEC/hRPE has been developed as an appropriate in vitro tool to gain fundamental understanding of the functional relationship between RPE/BM/choroid as shown in Fig. 1.

## Main text

### Methods

#### Isolation of hRPE and hCEC

Three eye globes used for the study include 32- and 35-years old males and 42-years old female donors. The eyes were collected within 6 h from death and processing was done within 2 h of procurement. The eyecups were incubated in PBS with antibiotic solution containing penicillin/streptomycin (Invitrogen). For isolation explant culture technique from Zhu et al. [3] with slight modifications was adapted. Briefly, anterior segments, the vitreous and retina were carefully removed in a sequential manner without disturbing the RPE layer. Eye globe was washed with PBS containing antibiotic solution (1:100) and floral sectioning was done with each section placed upside down on fibronectin (10 µg/ml dissolved in 1XPBS, sigma) coated 6 well plate. Then the sclera was removed leaving the RPE layer intact. 100 μl of endothelial growth medium (EGM-2) supplemented with growth factors was initially added on the explants and left undisturbed for about 4–5 h. Later, 1 ml of EGM2 medium was added and after overnight attachment, the explants were removed. The cultures were shifted to 0.1% gelatin coated flasks and grown in DMEM (HiMedia) with 10% FBS and antibiotic solution (1:100) after P0. hCEC isolation was done as we reported earlier [13]. Expression of epithelial cell specific cytokeratin-18 and endothelial specific vWF were analyzed by RT-PCR and immunocytochemistry.

#### ELISA and tube formation assay

The cleared medium from upper and lower chamber of the insert was used to detect the levels of pigment epithelial derived growth factor (PEDF) (Millipore, USA) and VEGF (R&D systems, USA) using respective ELISA kits as instructed by the manufacturer. The tube formation assay was done with hCEC cells seeded on the matrigel (Chemicon, USA) and pictures were taken with EVOS XLCore phase contrast microscope.

#### Cultures on transwell insert

Transwell inserts (0.4 µm polycarbonate 24 well, Corning Life sciences) were coated with fibronectin on both the sides. The epithelial cells were seeded with few drops of DMEMF-12/10% FBS for 4–6 h. Then the insert was washed to remove unattached cells and flipped back to seed hCEC on the upper side of the culture inserts with EGM-MV (without VEGF) (Promocell) for hCEC. The lower chamber was filled with 500 μl of DMEM/F-12/10% FBS and the upper chamber was filled with 200 μl of EGM-MV (without VEGF). The cells were allowed to grow for 11 days and media was replaced every 2 days.

#### Permeability flux with VEGF

Serum starvation medium with 1% FBS was given for 3 h to the bilayer culture and treated with 100 ng/ml of VEGF for 3 h. A final concentration of 1.25 mg/ml of 20 kDa FITC (Sigma) was added to the upper chamber of the insert. To counter check the VEGF effect, 0.125 mg/ml anti-VEGF agent (bevacizumab) was added at the end of 2 h [14]. During treatment, the plates were incubated at RT for 20 min and a basal fluorescence reading was taken using Spectromax M2^e^ (Molecular Devices, USA) with excitation at 485 nm and emission at 535 nm. Then the inserts were placed in incubator and every 1 h the reading was taken for 3–4 h. The apparent permeability coefficient (P_app_) was calculated using the following formula [15],

$${{\text{p}}_{{\text{app}}}} = \frac{{{\text{dQ}}}}{{{\text{dt}}}}\frac{1}{{{\text{A}}.{{\text{C}}_0}.60}}\left( {{\text{cm}}/{\text{s}}} \right)$$

where dQ/dt is the amount of FITC transported per minute (ng/min), A is the surface area of the filter (cm^2^), C is the initial concentration (ng/ml) and 60 is the conversion from minutes to seconds.

#### Statistical analysis

All the experiments were done in triplicates and the data were presented as mean ± SD. Wilcoxon signed rank test was done and the *p*-values of < 0.05 were regarded as significant.

### Results

#### Standardization of human primary cell cultures

**Fig. 2:**
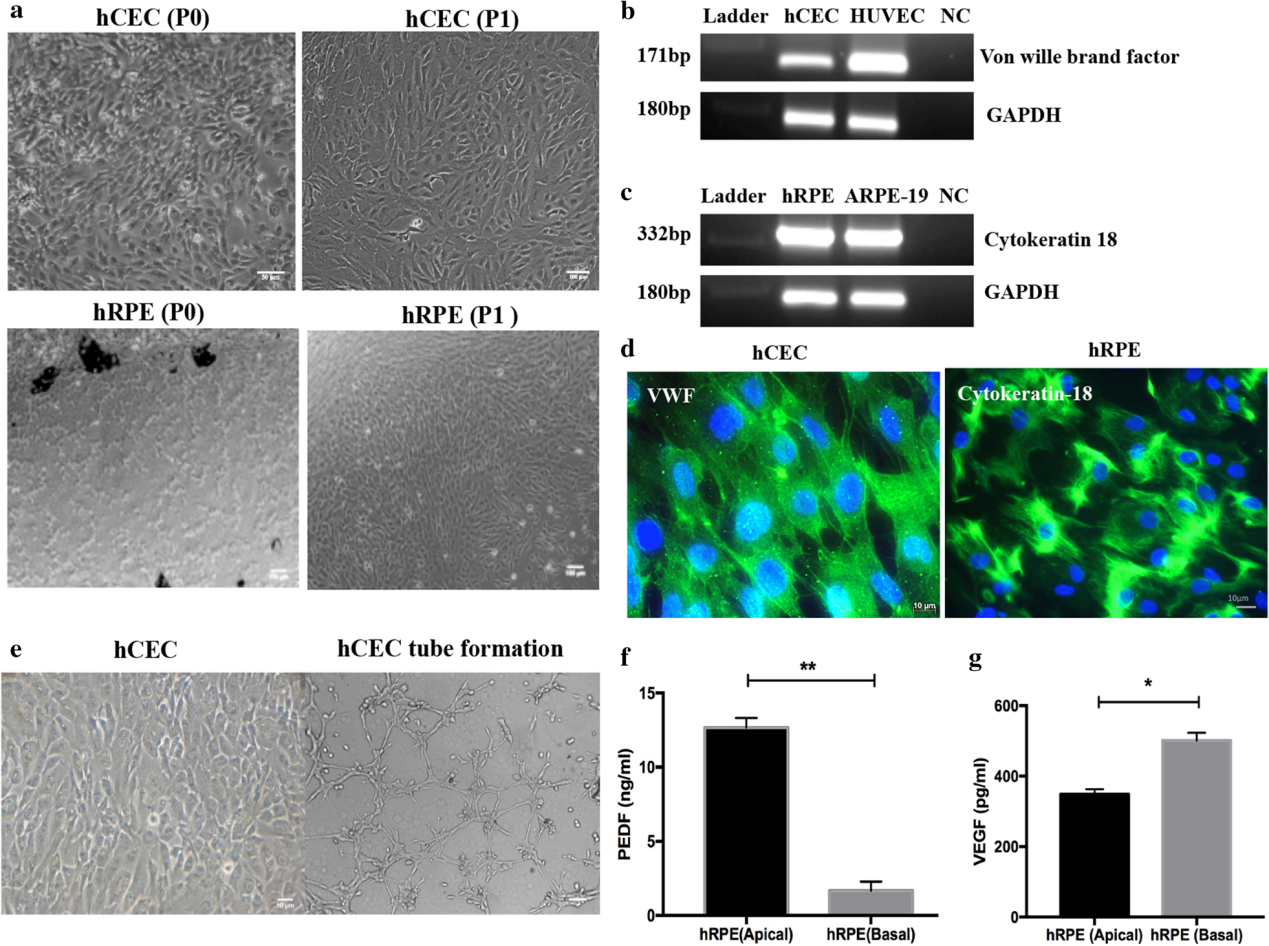
Images of human primary ocular cells. **a** Top left panel shows human microvascular choroidal endothelial cells (hCEC) freshly isolated (P0) and the first passage (P1) on the right side. Bottom left and right panels show the human retinal pigment epithelial (hRPE) cells at P0 and P1 respectively. **b** Semi-quantitative RT-PCR was done to show the presence of *von willebrand factor* transcripts in hCEC and HUVEC. **c** RT-PCR for *Cytokeratin*-*18* in the primary hRPE and ARPE19 cells was done. *GAPDH* was used as an internal control (n = 3). **d** Immunofluorescence staining of *von willebrand factor* in hCEC and *Cytokeratin*-*18* in hRPE (n = 3). **e** Tube formation assay was done on matrigel using hCEC. **f** *PEDF* levels in the medium from upper and lower chamber of insert grown hRPE was measured by ELISA, *p *= 0.006 (n = 3). **g** VEGF ELISA was performed in the medium taken from upper and lower chamber of hRPE *p *= 0.02 (n = 3)

Isolated hCEC culture initiated cobble stone appearance from 7 days after seeding (Fig. 2a top panel). The success of the culture greatly depended on age of the donor and time from harvest of the eyecups. The cells up to six passages were used for the study. We attempted to isolate hRPE by RPE Explant method [3] (Fig. 2a bottom panel) with different composition of medium and coating material. The yield was comparatively higher in the explant method with EGM-2 medium. Also, growth of the culture was faster, as early as 7–8 days in EGM-2 while DMEM-F12 showed growth after 11–14 days. The epithelial cells from RPE explants formed pigmented polygonal cells in aggregates in the pattern of honeycombs. After the P0 stage, the hRPE cells were completely grown in DMEM-F12 with 10% FBS. While, fibronectin coating of the culture dish enhanced the attachment of the cells at P0, both fibronectin and gelatin coated dishes showed similar levels of cell attachment for subsequent passages.

#### Marker identification and functional studies for the primary cells

The phenotype of the isolated primary cells was confirmed at different passages using RT-PCR and immunofluorescence staining of cell specific markers such as vWF as an endothelial specific marker for hCEC and cytokeratin 18 as an epithelial specific marker for hRPE (Fig. 2b–d). To assess whether the isolated hCEC were functional and homogenous, tube formation assay was done at different passages and the representative picture of hCEC forming tubes on matrigel is shown in Fig. 2e. Polarized growth of retinal epithelial cells is essential for proper barrier function, which alters the directionality of PEDF and VEGF secretion. PEDF secretion has shown to be increased in the apical side of polarized human fetal retinal pigment epithelial (hfRPE) cultures [16, 17]. Accordingly, the elevated level of PEDF in the lower chamber of the insert grown hRPE showed that the cells were polarized (Fig. 2f). Also the VEGF secretion in the hRPE basal side was high (Fig. 2g) and it is known to be necessary for the survival of the choriocapillaris [18, 19]. Thus, the isolated hCEC and hRPE cells expressed cell specific markers and displayed functional properties as previously attributed to them.

#### Validation of the bilayer models

**Fig. 3:**
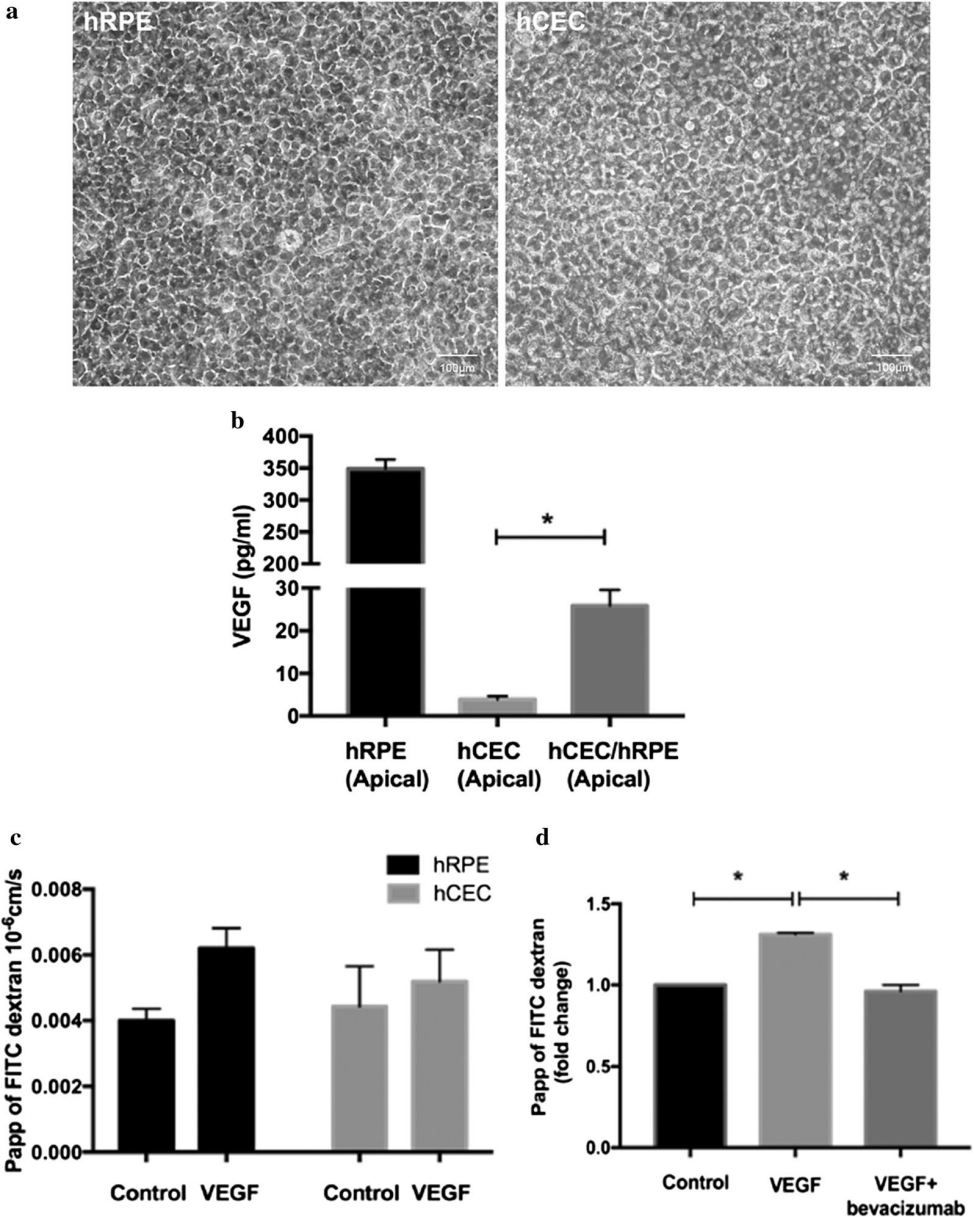
Validation of the bilayer model. **a** Phase contrast image of cells in the bilayer culture and the inlet picture shows morphology of the primary hRPE and hCEC grown on inserts. **b** VEGF ELISA was performed from the medium taken from upper chamber of hRPE, hCEC and hCEC/hRPE (*p *= 0.02) (n = 3). **c** Permeability coefficient of 20 kDa FITC dextran in hRPE and hCEC monolayer treated with 100 ng/ml VEGF (n = 3). **d** The bilayer of hCEC/hRPE was treated with 100 ng/ml VEGF and the permeability was calculated. At the end of 2 h anti-VEGF agent bevacizumab (0.125 mg/ml) was added and the permeability was measured for further 2 h, (n = 3)

The insert grown bilayer of hCEC/hRPE displayed typical cobblestone and honeycomb patterns of endothelial and epithelial cells respectively (Fig. 3a). In concurrence with previous study [20], the secretion of VEGF in the bilayer of hCEC/hRPE was significantly higher (22 pg/ml) than the hCEC monolayer (3 pg/ml) alone (Fig. 3b). Thus, we functionally characterized the bilayer model as a mimic for outer BRB.

#### Effect of VEGF on hCEC/hRPE

Pathologically high levels of VEGF is one of the major factors in disruption of outer BRB integrity [21–23] and thus, we wanted to investigate the effect of recombinant VEGF on hCEC/hRPE in comparison to individual monolayers. The monolayers of hRPE and hCEC showed increase in the paracellular flux with recombinant VEGF (Fig. 3c). Interestingly, hCEC/hRPE bilayer displayed a significant increase in the permeability (Fig. 3d). At the end of 2 h, anti-VEGF agent bevacizumab was added to the upper chamber to counteract the VEGF induced permeability. Thus, VEGF affected the bilayer permeability significantly.

### Discussion

In this study, we isolated primary hRPE and hCEC to establish a physiologically relevant RPE/choroid model using transwell insert (Fig. 1). The rationale was to choose a system that is simple, robust, as well as demonstrate a close resemblance to the physiological barrier of outer BRB. Most of the previous studies have been done in models accounting to the ease of cell preparation and availability of the source material. As Kuznetsova et al. [24], stated, the significance of primary cell cultures should be considered given the special clinical importance of such cells despite the difficulties in protocol optimization. In the similar line, majority of the cell systems used for diabetic retinopathy studies have been either of ‘nonhuman retinal origin or nonretinal human origin’ [25]. Therefore, isolation of human primary cells and creating relevant disease model system is necessary for proper understanding of the disease pathology.

The role of VEGF on the permeability of the outer barrier has been a controversy. VEGF treatment was shown to significantly increase the resistance in RPE cells [26] while another study showed that VEGF decreased the resistance in ARPE19 and primary porcine RPE [27]. Intriguingly, Peng et al. [28], reported that VEGF did not affect the permeability in hfRPE. Further, Hartnett et al. [29] showed that higher levels of VEGF in bovine RPE and bovine REC coculture could disrupt the barrier function. Using our model, we validated the findings of Harnett et al. that 100 ng/ml VEGF significantly increased the paracellular flux in hCEC/hRPE.

### Conclusions

Primary cells based bilayer model will be helpful in studying the crosstalk between RPE and choroid. It will also be useful in investigating the function of oBRB and any changes in the organization of tight junctions under pathological conditions. Further, we could study the effect of candidate molecules under controlled milieu. Inflammatory cell (macrophage) trafficking between choroid and retina could also be modeled in this system. Moreover, it would be useful for analyzing different drug delivery methods, to identify small molecules that can protect the barrier, and for in vitro toxicity analysis.

## Limitations

The hCEC/hRPE bilayer developed in this pilot study needs further characterization functionally, especially pertaining to barrier integrity measures such as transepithelial electrical resistance, expression of junction proteins etc. Further, the primary hRPE and hCEC cells would exhibit donor-to-donor variation. To overcome the problem of limited availability and donor variations of the primary cell cultures, human embryonic or induced pluripotent stem cell derived RPE cells could be used. Also, while we cultured cells upto 11 days, extended growth period is recommended for high polarity, which needs to be further optimized.

## Data Availability

Not applicable.

## Abbreviations

hCEC: human choroidal endothelial cells
hRPE: human retinal pigment epithelial cells
oBRB: outer blood retinal barrier
BM: Bruch’s membrane
vWF: von Willebrand factor
VEGF: vascular endothelial growth factor
FITC: fluorescein isothiocyanate
